# Arthropod Ectoparasites Have Potential to Bind SARS-CoV-2 via ACE

**DOI:** 10.3390/v13040708

**Published:** 2021-04-19

**Authors:** Su Datt Lam, Paul Ashford, Sandra Díaz-Sánchez, Margarita Villar, Christian Gortázar, José de la Fuente, Christine Orengo

**Affiliations:** 1 Institute of Structural and Molecular Biology, UCL, Darwin Building, Gower Street, London WC1E 6BT, UK; p.ashford@ucl.ac.uk; 2 Department of Applied Physics, Faculty of Science and Technology, Universiti Kebangsaan Malaysia, Bangi 43600, Selangor, Malaysia; 3 SaBio, Instituto de Investigación en Recursos Cinegéticos IREC-CSIC-UCLM-JCCM, Ronda de Toledo s/n, 13005 Ciudad Real, Spain; sandra.diaz@uclm.es (S.D.-S.); margaritam.villar@uclm.es (M.V.); Christian.Gortazar@uclm.es (C.G.); 4 Regional Centre for Biomedical Research (CRIB), Biochemistry Section, Faculty of Science and Chemical Technologies, University of Castilla-La Mancha, 13071 Ciudad Real, Spain; 5 Center for Veterinary Health Sciences, Department of Veterinary Pathobiology, Oklahoma State University, Stillwater, OK 74078, USA

**Keywords:** SARS-CoV-2, COVID-19, spike protein, ACE2, structural bioinformatics, parasite

## Abstract

Coronavirus-like organisms have been previously identified in Arthropod ectoparasites (such as ticks and unfed cat flea). Yet, the question regarding the possible role of these arthropods as SARS-CoV-2 passive/biological transmission vectors is still poorly explored. In this study, we performed in silico structural and binding energy calculations to assess the risks associated with possible ectoparasite transmission. We found sufficient similarity between ectoparasite ACE and human ACE2 protein sequences to build good quality 3D-models of the SARS-CoV-2 Spike:ACE complex to assess the impacts of ectoparasite mutations on complex stability. For several species (e.g., water flea, deer tick, body louse), our analyses showed no significant destabilisation of the SARS-CoV-2 Spike:ACE complex, suggesting these species would bind the viral Spike protein. Our structural analyses also provide structural rationale for interactions between the viral Spike and the ectoparasite ACE proteins. Although we do not have experimental evidence of infection in these ectoparasites, the predicted stability of the complex suggests this is possible, raising concerns of a possible role in passive transmission of the virus to their human hosts.

## 1. Introduction

Arthropods such as mosquitoes, flies, lice, fleas, ticks and mites infest humans, wildlife and domestic animals and constitute a global health problem [1,2]. Ectoparasitic blood-feeding arthropods directly affect host health and also act as vectors of pathogenic bacteria, parasites and viruses [2]. Insect and arachnid ectoparasites represent a major burden for human and animal health worldwide and novel interventions are required for the control of ectoparasite infestations and transmission of pathogens [1,3,4]. Despite the development and possibilities of multiple control strategies, vaccines constitute the most effective and sustainable intervention for the control of ectoparasite infestations and vector-borne diseases [3,5,6].

Host–vector–pathogen molecular interactions evolved as conflict and cooperation [7]. In this way, arthropods may benefit from host factors and pathogen-induced gene expression that favour tick feeding and fitness, midgut microbiota composition and changes in epigenetic regulatory mechanisms that facilitate tick survival under extreme environmental conditions, which results in evolutionarily conserved mechanisms that support pathogen infection with increased ectoparasite fitness and survival [7,8,9,10,11,12].

Arthropod ectoparasites may act as active biological transmission and/or passive transmission vectors of pathogenic microorganisms including viruses [13,14,15,16,17,18]. Viremia or infectious virus load detectable in vertebrate host circulating blood is required for biological transmission [13,18]. However, non-viremic arbovirus transmission is observed in viremia-free vertebrate hosts that play a role in disease epidemiology [13,18]. Biological vectors such as mosquitoes, mites, fleas, lice and ticks may carry pathogens that can multiply within their bodies for transmission to new hosts, usually by biting [14,19]. Mechanical vectors such as domestic flies, mosquitoes and ticks can collect infectious agents from contaminated objects, fluids and tissues and carry them on the outside (body exoskeleton, feet or mouth parts) or inside of their bodies and transmit them through physical contact with host body, food or drink [14]. In some vector–host–pathogen interactions, pathogens are transmitted both biologically after completing a life cycle in the vector and passively by intrastadial transmission (e.g., *Amblyomma hebraeum*–wild ungulates, domestic ruminants–lumpy skin disease virus, LSDV [20]) or from contaminated mouthparts to susceptible infested hosts (e.g., cat flea *Ctenocephalides felis*–cats, dogs, opossums, raccoons, rodents, humans–*Rickettsia felis* [21] and fly, *Stomoxys calcitrans*–cattle–*Anaplasma marginale* [22,23,24]). In other cases, only passive transmission through blood–meal regurgitation may occur (e.g., *Ornithodoros moubata*–human–HIV-1 [25]).

The hosts susceptible to severe acute respiratory syndrome coronavirus 2 (SARS-CoV-2) have a role in the coronavirus disease 19 pandemic (COVID-19) and are also infested by multiple blood-feeding arthropod ectoparasites [26,27,28]. Natural infections with SARS-CoV-2 have been reported in several animal species with evidence of zoonotic (animal-to-human) and reverse zoonotic (human-to-animal) virus transmission [28]. Coronavirus-like organisms have been previously identified in tick *Ixodes uriae* [29] and in unfed cat flea *Ctenocephalides felis* [30], thus raising the question of the possible role of ectoparasite arthropod vectors on SARS-CoV-2 passive and/or biological transmission [30].

Recently, models for SARS-CoV-2 Spike (S)-angiotensin I converting enzyme 2 (ACE2) host receptor interactions support that animal species with close-to-human S-ACE2 interactions (e.g., great apes or ruminants) may constitute effective hosts for maintenance and zoonotic transmission of the virus while other animal species with low S-ACE2 interaction capacity (e.g., cats or pigs) may be susceptible to reverse zoonotic transmission with low risk for human infection [31]. Additionally, the role of integrins also found in ectoparasite vectors [32] as co-receptors for SARS-CoV-2 and flavivirus cell attachment may also affect animal host susceptibility to infection [33,34]. Host- and virus-derived factors continue to be key drivers of the pandemic [35]. Based on the role of arthropod ectoparasites in pathogen passive and/or biological transmission and available preliminary evidence [29,30], the possible role of arthropod vectors in SARS-CoV-2 transmission needs to be considered.

We performed a number of protein structural and binding energy calculations to assess one of the steps in arthropod–pathogen interactions that may be associated with the risks of possible ectoparasite vector transmission. Since the complex formed between SARS-CoV-2 Spike protein and the human ACE2 receptor has been structurally determined (PDB code 6M0J [36]), we assessed whether the similarity of ectoparasite ACE proteins to human ACE2 was sufficient to model the SARS-CoV-2 Spike:ACE complex and assess the impacts of any residue mutations in ectoparasite ACE on the stability of this complex. We also performed detailed structural analyses to explore and characterise the possible structural mechanisms affecting complex stability. Our data suggest that there is sufficient similarity between ectoparasite ACE and human ACE2 protein sequences to build good quality 3D-models of the SARS-CoV-2 Spike:ACE complex to assess the impacts of ectoparasite mutations on complex stability. For several species where there were genomic data available (e.g., water flea, deer tick, body louse), our analyses showed no significant destabilisation of the SARS-CoV-2 Spike:ACE complex, suggesting these species would bind the viral Spike protein. In support of the stability calculations, we provide structural rationale for interactions between the viral Spike and the ectoparasite ACE proteins.

Our analyses of complex stability employed a platform previously established to analyse possible SARS-CoV-2 infection in a wide range of animal species. This earlier work allowed us to establish thresholds on whether changes in stability of the SARS-CoV-2 Spike:ACE2 complex, caused by residue mutations in the animal, were likely to affect infection. We have applied the same computational platform and thresholds to assess changes in stability of the SARS-CoV-2 Spike:ACE complex in ectoparasites, some of which are blood-feeding arthropod ectoparasites. Although we do not have experimental evidence of infection in these ectoparasites, the predicted stability of the complex suggests this is possible, raising concern of a possible role in passive transmission of the virus to their human hosts.

## 2. Materials and Methods

### 2.1. Sequence Data

We obtained ACE protein sequences in fruit fly (*Drosophila melanogaster*; Uniprot ID: Q10714), water flea (*Daphnia pulex*; E9GU43), water flea order (*D. pulex*; A0A162PAD4), body louse (*Pediculus humanus corporis*; E0VAB8), deer tick (*Ixodes scapularis*; A0A4D5RPS5), and common tick (*Ixodes ricinus*; A0A0K8R3C7). We used NCBI BLAST v.2.6 [37,38] to align protein sequences to the human ACE2 sequence (Q9BYF1).

### 2.2. Structural Data

We used the structure of the SARS-CoV-2 spike protein (at 2.45 Å resolution (PDB ID 6M0J [36])) reference strain, bound to human ACE2 as a template to model the structures of the ectoparasite ACE bound to the SARS-CoV-2 reference (Wuhan-Hu-1) and the ectoparasite ACE protein bound to different strains of the SARS-CoV-2 with mutations at various sites in the RBD. 

Models were built by generating query–template alignments using HH-suite version 3 [39], which were then used as input to the MODELLER v.9.24 program [40,41]. To optimise the geometry of the complex and interface we used the ‘very_slow’ schedule for model refinement. Ten models were generated for each S-protein:ACE complex and we then selected the model with the lowest normalised DOPE score (nDOPE) [42], which reflects the quality of the model. Positive scores are likely to be poor models, while scores lower than −1 are likely to be native-like.

### 2.3. Identification of Residues Involved in Binding of Viral SARS-CoV-2 to Host ACE

In order to determine the impact of mutations occurring between the ectoparasite ACE sequences and human sequences, on the stability of the SARS-CoV-2 S:ACE complex, we identified key residues in the interface of these proteins. In addition to residues in ACE2 that contact the S-protein directly, we also included residues that are in the second shell, or are buried, and could influence binding, as previous studies showed that mutations in these positions correlated well with experimental data on changes in complex stability [43]. Therefore, we used the following sets of residues for our study:

Direct contact (DC) residues: These are in direct contact with the S-protein [36] identified by PDBe [44] and PDBSum [45]. We identified 20 residues.

Direct contact extended (DCEX) residues: We identified residues within 8Å of DC residues which are likely to be important for binding. We used detailed manual inspection of the complex, and also selected residues according to (i) evidence from deep mutagenesis studies [41], (ii) in silico alanine scanning analyses (using mCSM-PPI2 [46]), (iii) high evolutionary conservation of the residues identified by the FunFam-based protocol described above, i.e., residues identified with DOPS [47] ≥ 70 and ScoreCons [47] score ≥ 0.7, (iv) allosteric site prediction [48,49,50], and (v) sites under positive selection [51,52,53]. We also included residues identified by other related structural analyses, reported in the literature [36,43,54,55,56,57].

### 2.4. Measuring Change in Residue Chemistry for Mutations

For each ectoparasite, we computed the Grantham score [58] comparing the ectoparasite ACE to the human ACE2 in order to understand the chemical shift associated with mutations involving the interface binding residues. Grantham score calculates volume, polarity, and atomic composition differences between amino acids. A Grantham score ranges from 0 to 215; higher Grantham scores are considered more disruptive to interface stability. The sums of Grantham scores were obtained for both DC and DCEX residues. 

### 2.5. Measuring Changes in the Stability of the S-Protein:ACE Complex. Following Mutation

We calculated the change in stability of the S-protein:ACE complex using mCSM-PPI2 [46]. This method was used in a previous analysis to analyse the impacts of mutations on the stability of the S-protein:ACE2 complex and was verified using data from in vitro and in vivo studies [31]. mCSM-PPI2 exploits machine learning models that analyse graph-based signature vectors for each mutation to predict the binding energy. The signature vector encodes multiple features including atom-distance patterns in the wild-type protein, pharmacophore information, available experimental information, evolutionary information and energetic terms. The mCSM-PPI2 server (http://biosig.unimelb.edu.au/mcsm_ppi2/, accessed on 1 January 2021) was used for the simulations. 

We produced ectoparasite ACE: SARS-CoV-2 Wuhan-Hu-1 complexes, ectoparasite ACE: SARS-CoV-2 N501Y complexes, and ectoparasite ACE: SARS-CoV-2 K417N, E484K, N501Y complexes. Using the model of ectoparasite ACE: SARS-CoV-2 complex, we mutated the ectoparasite ACE interface residues back to the appropriate residues found in the human ACE2 structure using mCSM-PPI2 to obtain the ΔΔG value. Positive ΔΔG values reflect destabilisation of the complex by the mutation and negative values reflect stabilisation of the complex.

### 2.6. Structural Analysis

We also evaluated structural changes for all combinations of RBD mutations and receptor complexes. We performed manual inspection of these key regions and others identified from studies by other groups [36,43,54,55,56,57,59]. We used UCSF Chimera v1.15 [60,61] to render structural images and predict H-bonds and salt bridges. Since all the 3D models were built using the very slow refinement option in MODELLER v9.24 [40,41], side-chain rotamers had been optimised. However, we also relaxed the allowable H-bond angle constraint to identify possible H-bonds at key hotspot residues. 

### 2.7. Phylogenetic Analyses of Cytochrome Oxidase Subunit 1

We performed a phylogenetic analysis using nine cytochrome oxidase subunit 1 (COI) nucleotide sequences downloaded from GenBank to generate a robust phylogenetic tree (GenBank Accession Numbers: JX150976; KY322738; MG432684; KX360345; MK140087; LC029557; MK905394; KX054334; outgroup: JF871600). The nucleotide sequences were aligned with MAFFT [62] using Geneious Prime v2.2 software [63] (https://www.geneious.com, accessed on 1 January 2021). Further, jModelTest2 was computed using the AIC (Akaike Information Criteria) to select the best nucleotide substitution model [64]. Genetic distance was computed using General Time Reversible model and gamma distributed rate variation among sites (GTR + G) [65]. The phylogenetic tree was inferred according to the Maximum Likelihood method with 100 bootstrap replicates using Maximum Likelihood in MEGAX software [66,67]. Phylogenetic tree annotation and visualization was performed using FigTree v1.4.4 (http://tree.bio.ed.ac.uk/, accessed on 1 January 2021).

### 2.8. Methods for Pan-Taxonomic Metazoan Comparison Analysis

To analyse sequence relationships across the tree of life, we include 24 pantaxonomic compara species found in the Ensembl Metazoa database [68]. However, we found ACE sequences for only 17 species. We compared these ACE sequences with human ACE2 sequences using NCBI BLAST v.2.6 [37,38]. We also used EMBOSS Needle [69] to calculate their sequence similarity with human ACE2 DCEX residue. A phylogenetic tree of invertebrate metazoan ACE DCEX residues was inferred using the Neighbour Joining method and BLOSUM62 substitution matrix.

## 3. Results

### 3.1. Comparison of Ectoparasite ACE with Human ACE2 Sequences

We compared the ectoparasite ACE with human ACE2 sequences using BLAST and found relatively high levels of sequence similarity (>35% in all cases), sufficient enough to model the structures for these proteins on the human ACE2 structure (Table 1).

### 3.2. How Many Mutated DCEX Residues Are There in the Interface and How Strong Are the Chemical Shifts?

We calculated the number of mutated DCEX residues (direct contact residues and residues within 8 Å of direct contact residues likely to influence binding, see Materials and Methods) and the Sum Grantham score (chemical shift) for mutation of these residues between ectoparasite and human sequences (Table 2).

The number of mutations and differences in the chemical properties of the mutated residues, compared to the human residue, are quite large. However, our previous analyses contrasting a wide range of animal ACE2 proteins with human ACE2 demonstrated that changes of this order did not necessarily disrupt binding of CoV2: Spike with host ACE2 protein [31].

### 3.3. Modelling the Structure of the SARS-CoV-2 Spike:ACE Complex. for the Ectoparasites (Based on the Wuhan-Hu-1 Strain) and Calculating the Change in Stability of the Complex. Compared to Human

In order to explore whether the changes between the ectoparasite ACEs and the human ACE2 would be likely to destabilise the Spike: ACE2 complex, we modelled the 3D structure of the Wuhan-Hu-1 S-protein: ectoparasite ACE complex using our FunMod modelling platform [70,71]. We used PDB 6M0J as the template. The quality of the models is given by the normalised DOPE scores shown in Table 3 below. It can be seen that the models all have nDOPE scores less than −1, indicating good quality models [40].

For each complex, the change in stability of the complex, ΔΔG, was predicted by mutating the ectoparasite ACE interface residue to the appropriate residue in the human ACE2. In our previous analysis, any animals with predicted ΔΔG <= 3.72 were considered to be at risk as these values correlated well with experimental evidence for infection [31]. Table 4 shows the ΔΔG values when considering direct contact (DC) residues and also for direct contact plus extended residues (DCEX, i.e., residues within 8A of the DC residues). Red values highlight those indicating low destabilisation and therefore risk of infection.

### 3.4. Structural Analyses of the Changes in the Interface of the SARS-CoV2 Spike: ACE Complex. (Wuhan-Hu-1 Strain) Relative to the Human Complex

We used the LIGPLOT program of PDBsum [72] to examine possible residue interactions between the Spike S protein and the ACE proteins, contrasting interactions in human proteins with those for the ectoparasite proteins. Several studies have highlighted the importance of key interaction sites between RBD of Spike S protein and ACE2, in particular, three sites on the interface: hydrophobic pocket, hotspot-353 and hotspot-31 [73]. These sites have previously been identified [74] as key to understanding why the SARS-CoV-2 S-protein binds to human ACE2 with high affinity and how the viral S-protein has evolved to bind with much higher affinity to human ACE2 than SARS-CoV [36,63,75].

**Figure 1:**
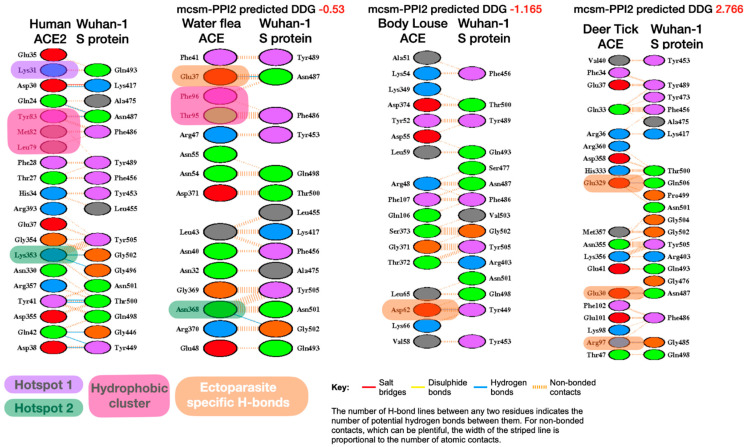
Comparison of LigPlot predicted bonding interactions for Wuhan-Hu-1 strain with human, water flea, body louse and deer tick. Corresponding residues in each species with key human interface hotspots (Hotspot 1, Hotspot 2) and the hydrophobic pocket are indicated if they are predicted to be involved in interface interactions. Novel ectoparasite H-bond residues also shown (pale orange boxes).

It can be seen from Figure 1 below that a number of hydrogen bonds have been lost in the ectoparasites, including some for these key interaction sites. However, for some of the ectoparasites (i.e., water flea, body louse and deer tick) there has been an increase in non-bonded contacts, and these are distributed across the whole interface.

**Figure 2:**
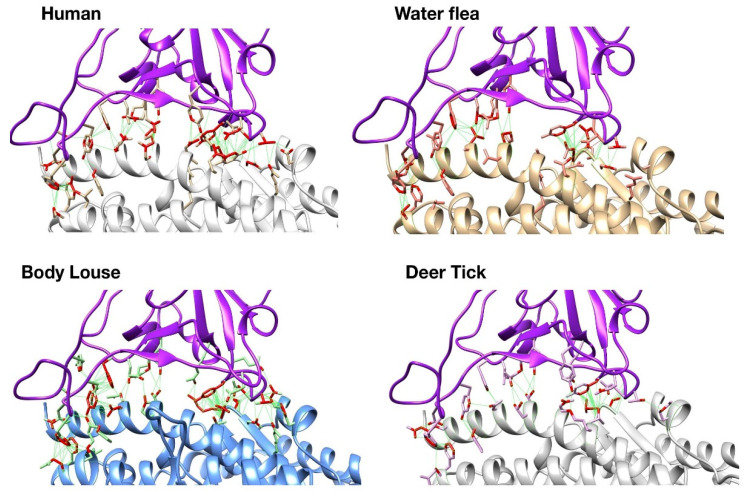
Structures of human and modelled ectoparasite SARS-CoV-2 S-protein: ACE complexes.

UCSF Chimera was also used to visually inspect these changes on the 3D structures of the complexes. Whilst human S-protein:ACE2 interface has close contacts across the whole interface including nine predicted H-bonds, the ectoparasites have very few hydrogen bonds (Figure 2). There are other structural changes that could also contribute to differences in the overall stability. However, as mentioned above these may be compensated for by the increase in non-bonded contacts. Deer tick and water flea have ~60 non-bonded contacts compared to ~80 in human, whilst body louse has ~130 across the whole interface, which may explain the small increase in stability of the S-protein:ACE complex relative to human. These ranges of ΔΔG value are probably within the error range for this method. Nevertheless, there is some structural rationale to support the suggestion that the complexes formed for these ectoparasites could be stable enough to support binding.

### 3.5. N501Y Mutant

In order to explore the likely impacts of the recent N501Y variant strain of SARS-CoV-2, we also modelled the N501Y S-protein: ectoparasite ACE using our modelling platform. We used 6M0J as the template. Again, good quality models were obtained for all the ectoparasite S-protein:ACE complexes (Table 5).

For each complex, ΔΔG values were predicted by mutating the ectoparasite ACE interface residue to the appropriate residue in the human ACE2 (Table 6). As in our previous analysis, any animals with predicted ΔΔG <= 3.72 are considered to be at risk. Interestingly, we observe that this mutation in the spike RBD domain results in a slightly increased stability of the complex for body louse, calculated over the DCEX residues (ΔΔG-1.92 N501Y versus-1.17 Wuhan-Hu-1).

**Figure 3:**
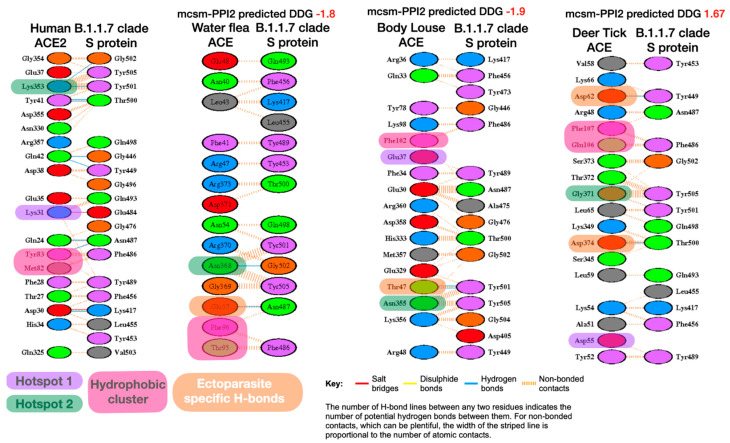
Comparison of LigPlot predicted bonding interactions for B.1.1.7 clade with human, water flea, body louse and deer tick. Corresponding residues in each species with key human interface hotspots (Hotspot 1, Hotspot 2) and the hydrophobic pocket are indicated if they are predicted to be involved in interface interactions. Novel ectoparasite H-bond residues also shown (orange boxes). In body louse, the ectoparasite-specific H-bond between Thr47 and Tyr501 is indicated as an equivalent to the important ‘Hotspot 2’ found in human spike:ACE2 interface.

Again, LIGPLOT analysis and visual inspection of the structures was performed to explore the rationale for the stability results (Figure 3).

**Figure 4:**
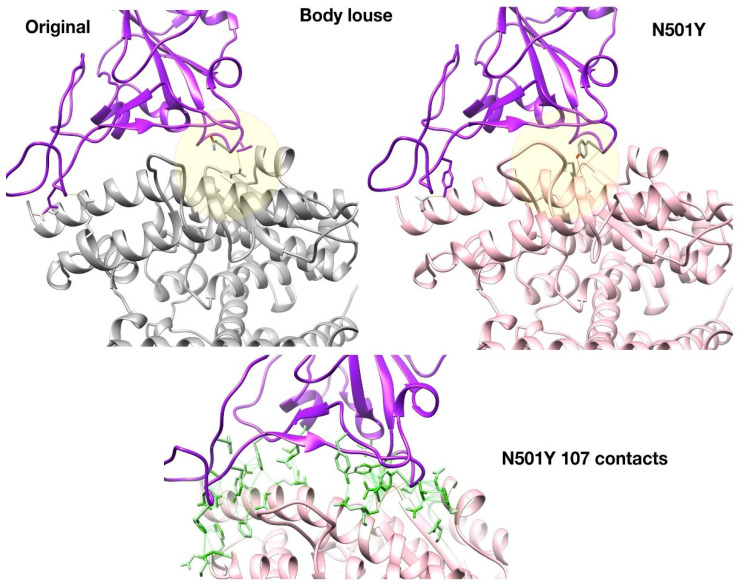
Structures of N501Y mutant show increased H-bond stabilisation in body louse.

Figure 4 shows that the N501Y mutation is predicted to more readily form H-bonds in part of the Spike:ACE interface in body louse (yellow circle) due to favourable geometry and the proximity of Tyrosine hydroxyl group to ACE interface residues. This H-bond (Tyr501-Thr47) enabled by the N501Y mutation stabilises part of the interface in body louse by binding to the ACE2 residue Thr47. It is within 5A of the ‘Lys-353 hotspot’ identified as an important component of the Spike:human-ACE2 interface [73]. In body louse, Lys353 is replaced by Asn355; it is therefore possible that Tyr501-Thr47 provides an alternative H-bond component to the interface stability near this ‘hotspot353’ region.

Whilst there is a slight reduction in the number of non-bonded contacts relative to the Wuhan-Hu-1 strain, the N501Y Body Louse still has >100 non-bonded contacts across the interface combined with H-bond stabilisation at two distinct patches.

### 3.6. K417N, E484K, N501Y Mutants

We also explored the impact of the combined variants observed in recent strains from South Africa and Brazil by modelling K417N, E484K, N501Y S-protein: ectoparasite ACE using our modelling platform (Figures S1 and S2, Tables S1 and S2). As with the N501Y mutant, we found that the combined variants had the effect of further stabilising the complex for body louse, water flea and the common tick. Structural analyses indicate that H-bonding in this strain could be behind the relatively high interface stability predicted for water flea and body louse according to the ΔΔG values. Limited H-bonds were predicted for deer tick, in agreement with the overall weaker predicted ΔΔG for this interface.

### 3.7. Phylogenetic Analysis

**Figure 5:**
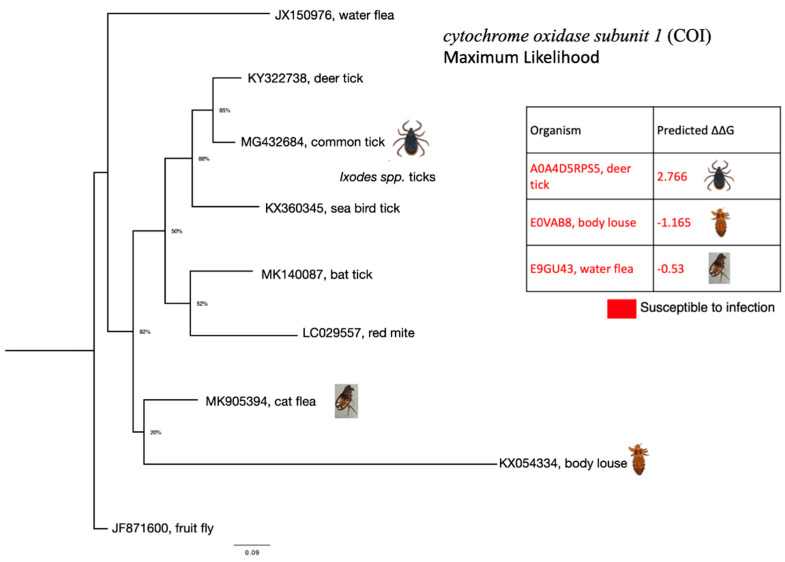
Phylogenetic tree of ectoparasite cytochrome oxidase subunit 1 (COI) nucleotide sequences. The phylogenetic tree was inferred according to the Maximum Likelihood method. Genetic distance was computed using General Time Reversible model and gamma distributed rate variation among sites (GTR + G).

As mentioned above, coronavirus-like organisms have been previously identified in sea bird tick *Ixodes uriae* [29] and in unfed cat flea *Ctenocephalides felis* [30]. We were unable to obtain both sea bird tick and cat flea ACE sequences. Therefore, to understand the relationship between these ectoparasites we produced a phylogenetic tree using cytochrome oxidase subunit 1 (COI) sequences. Figure 5 below demonstrates that cat flea COI belongs to the same clade as body louse COI. Sea bird tick COI belongs to the same clade as deer tick and common tick COIs. Other ectoparasite species in the tree could not be analysed using our protocol since their ACE sequences were also unavailable.

### 3.8. Pan-Taxonomic Metazoan Comparison

We examined whether the significant similarity of ectoparasite ACE to human ACE2 (~40% sequence identity or higher) and the stable complex formed with SARS-CoV-2 Spike protein suggested selection pressure on the virus to evolve binding affinity to both animal hosts and their insect hosts. To do this, we performed a pantaxonomic analysis to determine the ΔΔG values for a range of invertebrate species (Figures S3–S7, Tables S3–S6). It can be seen from Figure S6 that other invertebrates also have low ΔΔG values, including several species (octopus, African spider, honeybee) that are not ectoparasites, suggesting that the stability of the SARS-Cov-2 S-protein:ACE complex is not a result of selection pressure on the virus to evolve binding affinity to both animal hosts and their insect hosts.

## 4. Discussion

We have been able to obtain the protein sequences of ACE proteins and model the structural complexes of CoV2 Spike S-protein:ACE proteins in five ectoparasite species. The levels of sequence similarity between the ectoparasite sequences and the human ACE2 sequence suggested the possibility of similarity in the protein interface. Our energetic analyses, which examined the likely impacts of mutations between the human/ectoparasite sequences, showed a slight destabilisation in the complex for deer tick and common tick, whilst for the water flea and the body flea slight increases in stability of the complex were observed (ΔΔG < 0). All the ΔΔG values measured were small and within the range of values observed in a previous study of animal hosts [31] as being likely to be associated with susceptibility to infection. As mentioned above, we are not suggesting infection of the parasites as there are currently no experimental data to support that but our data suggest that the virus would be able to attach to membrane-associated proteins (e.g., ACE) on the ectoparasite cell surface and that this may provide a mechanism for passive transmission of the virus.

Based on the results of this study and previous evidence on the role of arthropod vectors in biological and passive virus transmission [76] and the presence of coronaviruses in ticks and cat flea [29,30], our hypothesis is that insects and arachnids could have a potential role in SARS-CoV-2 transmission. Current evidence does not support SARS-CoV-2 vector biological transmission. However, the prolonged environmental stability of the virus [77,78] and putative interactions with vector ACE2 and integrin proteins suggest a possibility for SARS-CoV-2 passive transmission. Virus passive transmission may occur via contact with SARS-CoV-2-contaminated substrates and surfaces and/or through contaminated mouthparts, blood-meal regurgitation or mechanisms similar to RNA interference (RNAi) [79].

**Figure 6:**
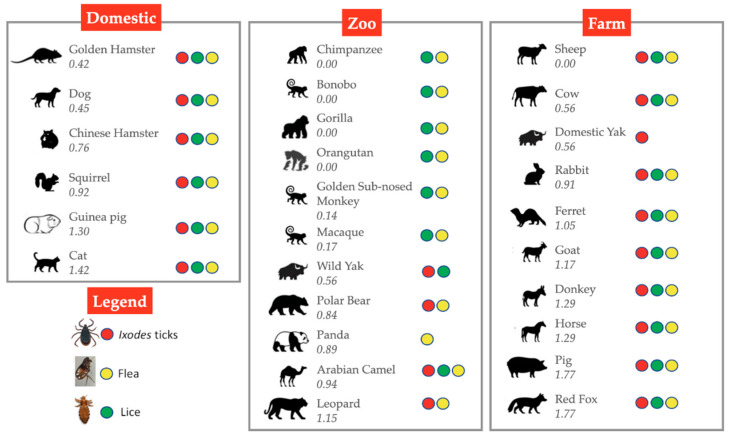
Animal hosts for ectoparasites. These animal hosts may be in contact with humans in domestic, agricultural or zoological settings. Numbers represent the predicted change in binding energy (ΔΔG) of the S-protein:ACE2 [31]. Supporting references [80,81,82,83].

Infestations with blood-sucking arthropod ectoparasite vectors have been documented in animal species reported or predicted to host SARS-CoV-2 as in humans (Figure 6) [80,81,82,83]. Furthermore, ACE and integrin alpha and beta proteins have been identified in cat flea exoproteome [30,84] and tick salivary glands and cement [85], thus supporting membrane exposure and secretion of these molecules. As documented in ticks and in other arthropods [79,86], RNAi is based on the entry of exogenous or viral double-stranded RNA (dsRNA) into the cytoplasm through injection, feeding or virus production of dsRNA resulting in transstadial and inherited (transovarial) RNAi. Accordingly, it is possible that SARS-CoV-2 may be acquired by feeding ectoparasites and through interactions with ACE and integrins it may persist not only in contaminated mouthparts, but also inside the vector for transmission to susceptible hosts by blood-meal regurgitation during secondary feeding or after transstadial transmission or inherited virus RNA. In support of the possibility of inherited virus, coronavirus-derived proteins and RNA were identified in adult unfed cat flea [30].

**Figure 7:**
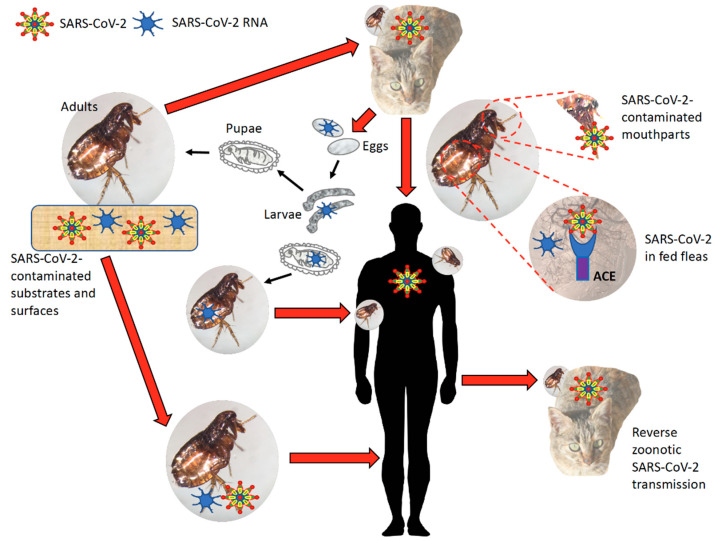
Proposed role of arthropod vectors in SARS-CoV-2 passive transmission. Using flea as a model, coronavirus passive transmission could occur via contact with SARS-CoV-2-contaminated substrates and surfaces. Fleas can feed on SARS-CoV-2-infected animal hosts and virus transmission to humans occurs via contaminated mouthparts, blood-meal regurgitation or inherited virus RNA. Similar to RNAi mechanisms, virus RNA may be also transmitted transovarially and transstadially. Reverse zoonotic SARS-CoV-2 transmission may also occur with arthropod passive vectors.

Using the flea as a model arthropod vector [87], SARS-CoV-2 passive transmission may occur via different routes (Figure 7). If proven true in experimental animal trials, these results add a new player to the SARS-CoV-2 persistence and transmission and possible selection of new virus variants.

As with our previous studies of complex stability in mammalian hosts [31], it is important to bear in mind the caveats associated with these types of studies. Although the computational protocol we used (exploiting 3D modelling and mutation studies by mCSM-PPI2) has been validated by experimental results obtained for a number of animal hosts, some of these studies captured in vitro rather than real life data. However, recent reports do support more of our predictions (experimental studies, [6,26,88,89,90,91,92], real-life data, [93,94,95,96]) further validating this approach. In addition, despite concerns raised on possible SARS-CoV-2 passive transmission by insects [97,98,99], recent studies have shown a failure of SARS-CoV-2 to infect or transmit in mosquitos, supporting our calculations on complex stability for this ectoparasite [100,101]. The stabilisation values reported in this manuscript are small, within the range of error for the methods used, suggesting that it is likely that the SARS-CoV-2 S-protein:ACE complex could form in ectoparasites. Although our structural analyses revealed that some key interactions in the interface are lost in the ectoparasite hosts this seems to be compensated by increased numbers of non-covalent interactions between residues in the binding partners. Furthermore, horseshoe bats which have been considered as a putative reservoir host, are reported infected from in vitro experiments, despite considerable disruption in the interface and destabilization of the complex (ΔΔG 3.7). However, whilst supporting the likelihood of complex formation we reiterate that our results do not provide any evidence supporting infection in the ectoparasites. They do prompt some concern though, and suggest that further exploratory experimental studies would be valuable.

Experimental approaches to validate SARS-CoV-2 S-protein:ACE complex formation in ectoparasites and their possible in virus transmission could include (a) evaluation of coronavirus survival in alimentary tract and midgut tissues after contact with SARS-CoV-2 after feeding on infected hosts or exposure to virus-spiked medium [102], (b) artificial injection of SARS-CoV-2 (i.e., intrathoracic inoculation) and sampling at different time points for in vitro titration of virus levels [101], and (c) ability of SARS-CoV-2-exposed ectoparasites to transmit the coronavirus to exposed environments and susceptible experimentally infested animal hosts [102].

## Figures and Tables

**Table 1:** BLAST sequence identities of ectoparasite ACEs and human ACE2.

Organism	Sequence Identity	E-Value
A0A162PAD4, water flea order	40.1%	1.99 × 10^−155^
A0A4D5RPS5, deer tick	38.9%	1.30 × 10^−149^
A0A0K8R3C7, common tick	37.7%	5.29 × 10^−136^
E0VAB8, body louse	39.6%	3.63 × 10^−145^
E9GU43, water flea	39.6%	6.60 × 10^−159^
Q10714, fruit fly	36.3%	1.31 × 10^−139^

**Table 2:** Mutated direct contact (DC), DCEX residues and the Sum Grantham score of ectoparasite ACEs.

Organism	Number of Mutated DC Residues	Number of Mutated DCEX Residues	Sum Grantham Score
Water flea order	16	34	2795
Deer tick	16	34	2581
Common tick	17	34	2790
Body louse	14	33	2561
Water flea	15	34	2895
Fruit fly	15	35	2580

**Table 3:** Normalised DOPE scores of SARS-CoV-2 Wuhan-Hu-1 S-protein: ectoparasite ACE complexes.

Organism	nDOPE Score
Water flea order	−1.12
Deer tick	−1.26
Common tick	−1.09
Body louse	−1.03
Water flea	−1.11
Fruit fly	−1.10

**Table 4:** Predicted ΔΔG values of SARS-CoV-2 Wuhan-Hu-1 S-protein: ectoparasite ACE complexes for direct contact (DC) residues and also for direct contact plus extended (DCEX) residues. Ectoparasites are categorised according to risk of infection by SARS-CoV-2, with ΔΔG  ≤  3.72 being at risk (red), and ΔΔG  >  3.72 not at risk (blue).

Organism	Predicted ΔΔG (DC)	Predicted ΔΔG (DCEX)
Water flea order	5.99	7.43
Deer tick	1.60	2.77
Common tick	1.97	2.79
Body louse	−2.31	−1.17
Water flea	−1.33	−0.53
Fruit fly	5.88	4.16

**Table 5:** Normalised DOPE scores of SARS-CoV-2 N501Y S-protein: ectoparasite ACE complexes.

Organism	nDOPE Score
Water flea order	−1.15
Deer tick	−1.23
Common tick	−1.15
Body louse	−0.93
Water flea	−1.18
Fruit fly	−1.09

**Table 6:** Predicted ΔΔG values of SARS-CoV-2 N501Y S-protein: ectoparasite ACE complexes for direct contact (DC) residues and also for direct contact plus extended (DCEX) residues. Ectoparasites are categorised according to risk of infection by SARS-CoV-2, with ΔΔG  ≤  3.72 being at risk (red), and ΔΔG  >  3.72 not at risk (blue).

Organism	Predicted ΔΔG (DC)	Predicted ΔΔG (DCEX)
Water flea order	5.49	7.18
Deer tick	1.72	1.67
Common tick	1.25	0.24
Body louse	−2.17	−1.92
Water flea	−2.37	−1.81
Fruit fly	7.13	5.99

## Data Availability

The authors confirm that the data supporting the findings of this study are available within the article and its supplementary materials.

## Supplementary Materials

The following are available online at https://www.mdpi.com/article/10.3390/v13040708/s1, Figure S1: Comparison of LigPlot predicted bonding interactions for B.1.351 clade with human, water flea, body louse and deer tick, Figure S2: Structures of this triple mutant show enhanced H-binding in water flea and body louse, Figure S3: BLAST sequence identities of invertebrate metazoan ACEs and human ACE2, Figure S4: DCEX residue similarities of invertebrate metazoan ACE and human ACE2, Figure S5: Phylogenetic tree of invertebrate metazoan ACE DCEX residues, Figure S6: Predicted ΔΔG values of SARS-CoV-2 Wuhan-Hu-1 S-protein: invertebrate metazoan ACE complexes for direct contact plus extended (DCEX) residues, Figure S7: Predicted ΔΔG values of SARS-CoV-2 N501Y S-protein: invertebrate metazoan ACE complexes for direct contact plus extended (DCEX) residues, Table S1: Normalised DOPE scores of SARS-CoV-2 K417N, E484K, N501Y S-protein: ectoparasite ACE complexes, Table S2: Predicted ΔΔG values of SARS-CoV-2 N501Y S-protein: ectoparasite ACE complexes for direct contact (DC) residues and also for direct contact plus extended (DCEX) residues, Table S3: Invertebrate metazoan ACE sequences studied, Table S4: Mutated DC and DCEX residues and the sum Grantham score of invertebrate metazoan ACEs, Table S5: Normalised DOPE scores of SARS-CoV-2 Wuhan-Hu-1 S-protein: invertebrate metazoan ACE complexes, Table S6: Normalised DOPE scores of SARS-CoV-2 N501Y S-protein: invertebrate metazoan ACE complexes.
