# Supplementary material for: Arthropod Ectoparasites Have Potential to Bind SARS-CoV-2 via ACE
Source: Viruses. 2021 Apr 19;13(4):708. doi: 10.3390/v13040708 (PMC8073597; doi:10.3390/v13040708)
Supplement: Supplementary file 1 [file viruses-13-00708-s001.zip › viruses-1189319-supplementary.pdf]

Analyses of the K417N, E484K, N501Y mutant

We also explored the impact of the combined variants observed in recent strains from South Africa and Brazil by modelling K417N,E484K,N501Y S-protein: Ectoparasite ACE using our modelling platform. We used 6M0J as the template.

Table S1. Normalised DOPE scores of SARS-CoV-2 K417N,E484K,N501Y S-protein: ectoparasite ACE complexes

Organism	nDOPE score
A0A162PAD4, water flea order	-1.08
A0A4D5RPS5, deer tick	-1.19
A0A0K8R3C7, common tick	-1.17
E0VAB8, body louse	-0.94
E9GU43, water flea	-1.10
Q10714, fruit fly	-1.13

Energetic analyses

Again, for each complex, ΔΔG were predicted by mutating the animal ACE2 interface residue to the appropriate residue in the human ACE2. As in our previous analysis, any animals with predicted ΔΔG <= 3.72 are considered to be at risk.

Table S2. Predicted ΔΔG values of SARS-CoV-2 N501Y S-protein: ectoparasite ACE complexes for direct contact (DC) residues and also for direct contact plus extended (DCEX) residues.

Organism	Predicted ΔΔG (DC)	Predicted ΔΔG (DCEX)
A0A162PAD4, water flea order	5.35	7.08
A0A4D5RPS5, deer tick	1.45	2.06
A0A0K8R3C7, common tick	-0.11	-0.16
E0VAB8, body louse	-2.54	-1.44
E9GU43, water flea	-2.51	-1.59
Q10714, fruit fly	5.15	4.36

Structural analyses

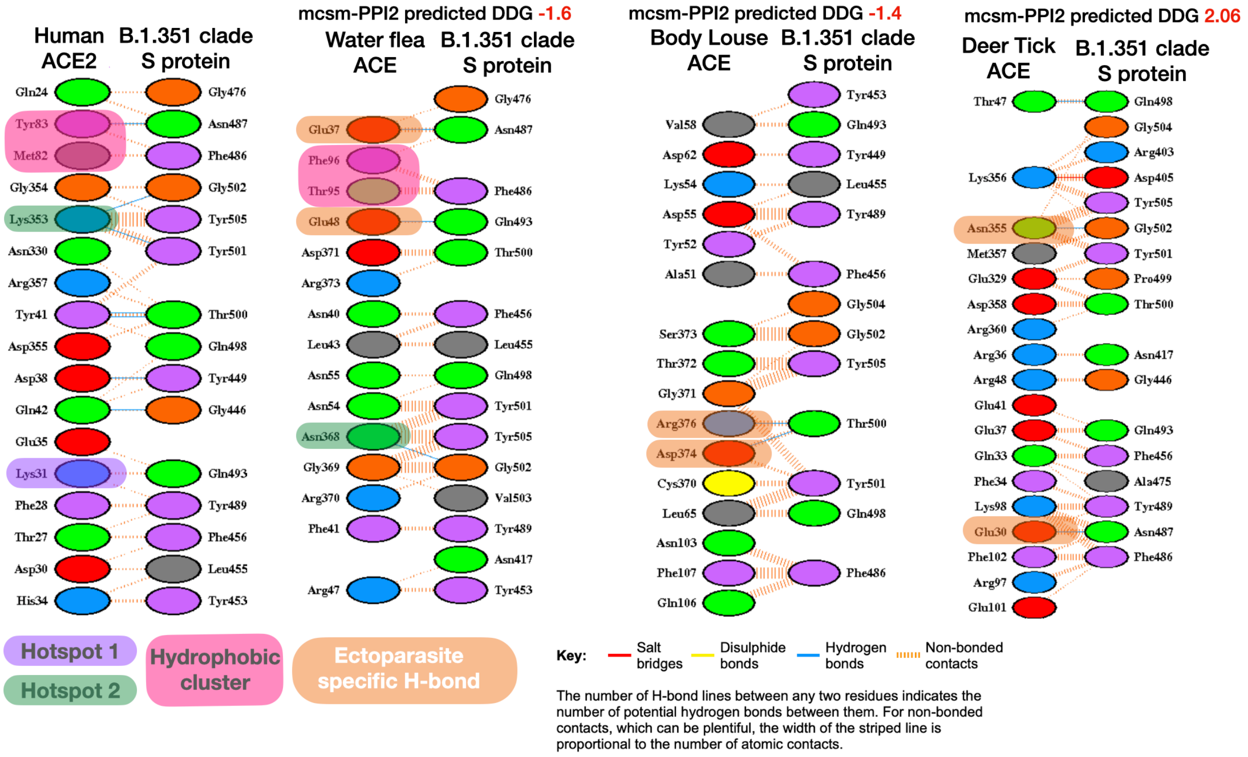

Figure S1. Comparison of LigPlot predicted bonding interactions for B.1.351 clade with human, water flea, body louse and deer tick. Corresponding residues in each species with key human interface hotspots (Hotspot 1, Hotspot 2) and the hydrophobic pocket are indicated if they are predicted to be involved in interface interactions. Novel ectoparasite H-bond residues also shown (orange boxes).

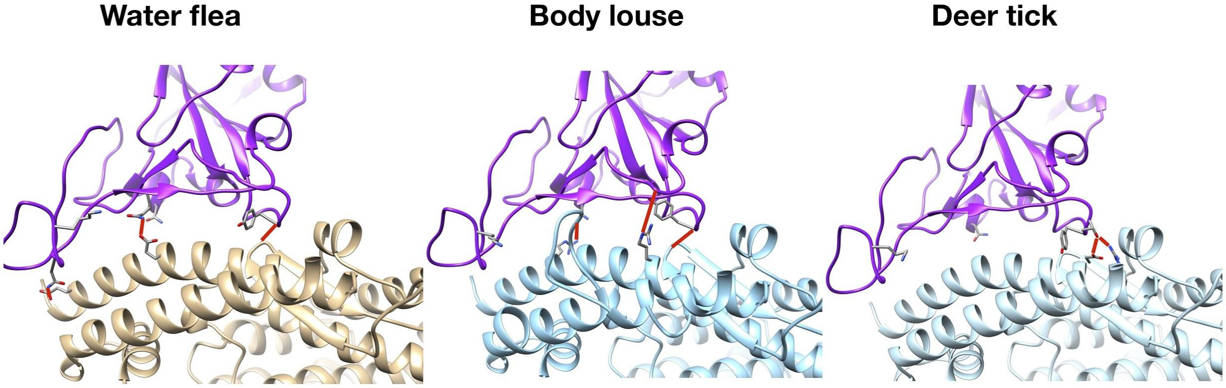

Figure S2. Structures of this triple mutant show enhanced H-binding in water flea and body louse. H-bonds identified from structural models suggest stabilisation of water flea and body louse interface Left In Water flea three H-bonds (Asn487 - Glu37; Gln493 - Glu48; Gly502 - Asn368) occur spread across whole interface (including near the hydrophobic pocket region); Mid In body louse three H-bonds predicted (Asn417 - Arg36; Gly446 - Arg48; Gly502 - Asn355); Right In deer tick only a single 'hotspot' is predicted to involve H-bonds (Thr500 - Asp374; Thr500 - Arg376).

In water flea H-bond Gln493 - Glu48, the Glu48 is equivalent to Glu35 in human ACE2, a critical interface hotspot. In body louse, one H-bond (Asn417 - Arg36) is mediated directly by the K417N mutation.

Pan-taxonomic metazoan comparison

We examined whether the significant similarity of ectoparasite ACE to human ACE2 (~40% sequence identity or higher) and the stable complex formed with SARS-CoV-2 Spike protein, suggested selection pressure on the virus to evolve binding affinity to both animal hosts and their symbiotic insect hosts. To do this we analysed sequence relationships across the tree of life. There were a total of 114 invertebrate species found in the Ensembl Metazoa database. We only include pantaxonomic compara species. This gave 24 species. However, we found ACE sequences for only 17 species.

Table S3. Invertebrate metazoan ACE sequences studied

Sequence	Species
A0A067RH69	*Zootermopsis nevadensis* (Dampwood termite)
A0A087TSS0	*Stegodyphus mimosarum* (African social velvet spider)
A0A087ZN26	*Apis mellifera* (Honeybee)
A0A0L8HWW2	*Octopus bimaculoides* (California two-spotted octopus)
A0A1P6BV99	*Brugia malayi* (Filarial nematode worm)
A7SKY7	*Nematostella vectensis* (Starlet sea anemone)
B3RUA8	*Trichoplax adhaerens* (Trichoplax reptans)
D2A565	*Tribolium castaneum* (Red flour beetle)
E0VAB8	*Pediculus humanus subsp. corporis* (Body louse)
E9GU43	*Daphnia pulex* (Water flea)
Q10714	*Drosophila melanogaster* (Fruit fly)
Q7Q9W7	*Anopheles gambiae* (African malaria mosquito)
T1FT41	*Helobdella robusta* (Californian leech)
T1ISP9	*Strigamia maritima* (European centipede)
T1KX65	*Tetranychus urticae* (Two-spotted spider mite)
V4AKT8	*Lottia gigantea* (Giant owl limpet)
W4YHX1	*Strongylocentrotus purpuratus* (Purple sea urchin)

We compared these ACE sequences with human ACE2 sequences. The average sequence identity is 36% i.e. similar to that observed for the ectoparasites.

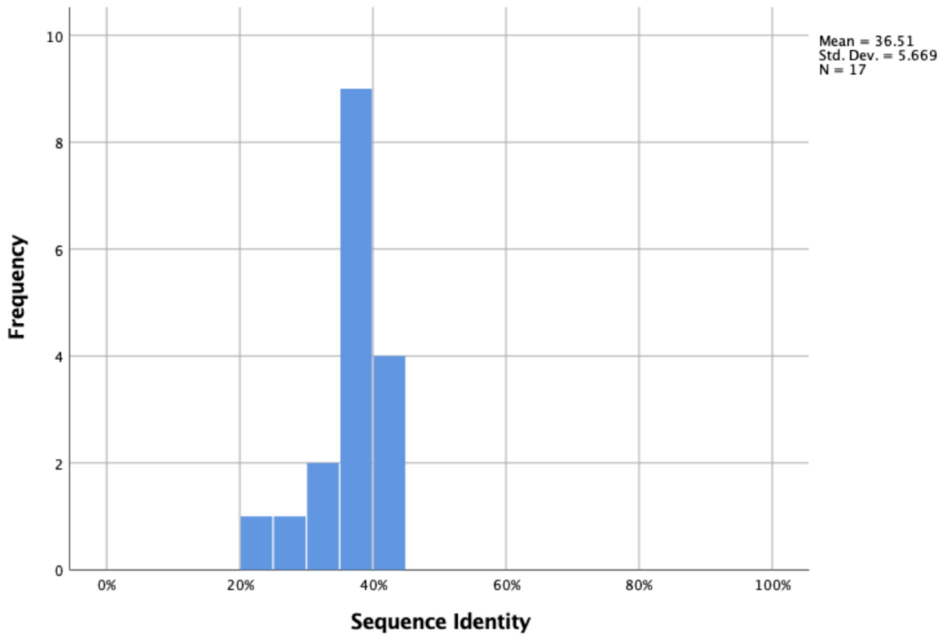

Figure S3. BLAST sequence identities of invertebrate metazoan ACEs and human ACE2.

For each sequence, we extracted the DCEX residues. Then, we used EMBOSS Needle to calculate their sequence similarity with human ACE2 DCEX residues. We removed 6 models that were missing > 10 DCEX residues. This gave 11 ACE sequences.

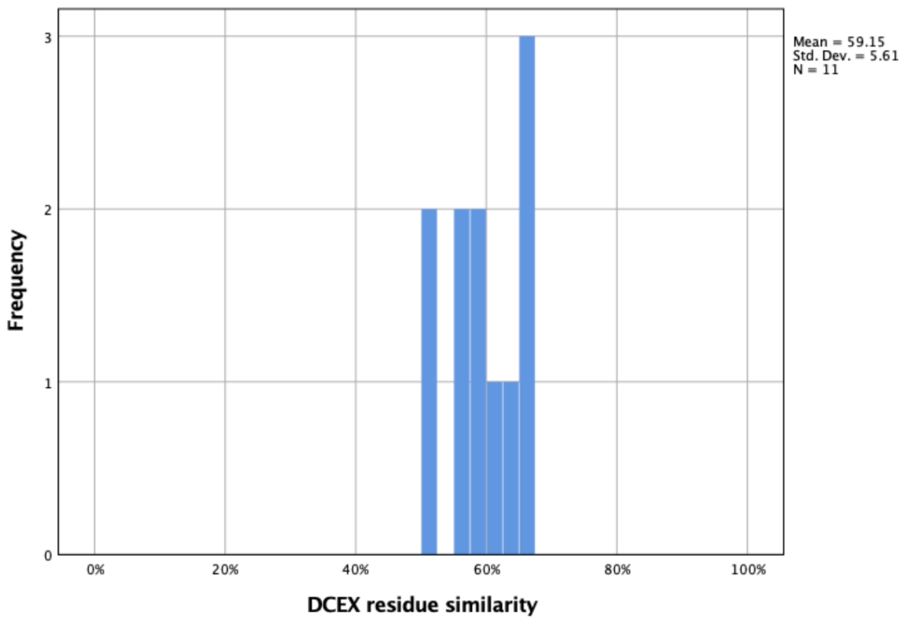

Figure S4. DCEX residue similarities of invertebrate metazoan ACE and human ACE2.

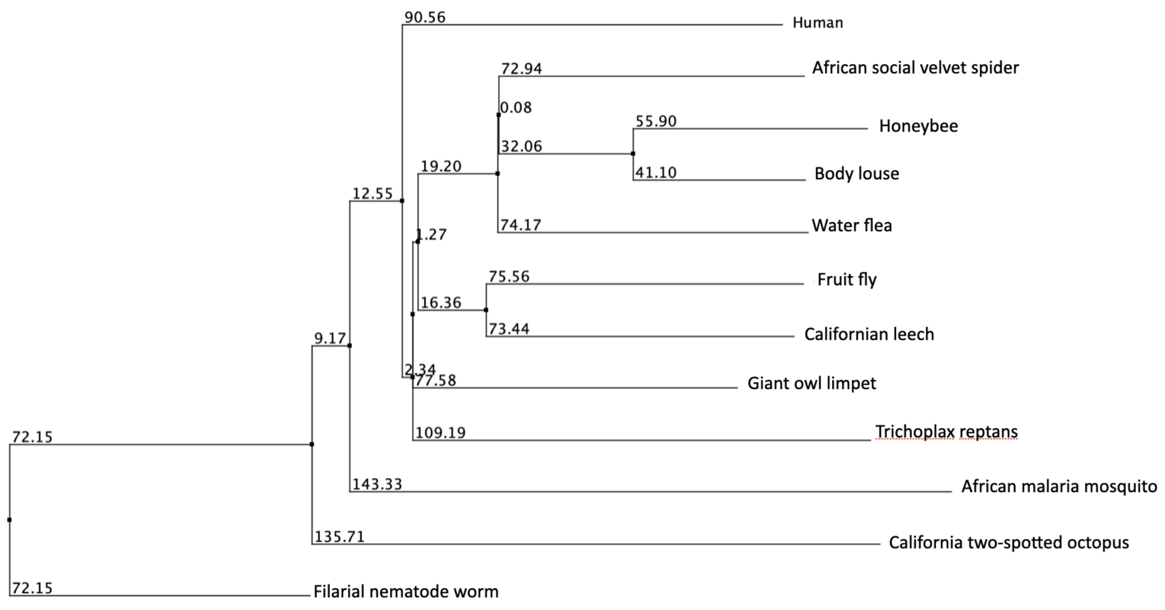

Figure S5. Phylogenetic tree of invertebrate metazoan ACE DCEX residues. The phylogenetic tree was inferred using the Neighbour Joining method and BLOSUM62 substitution matrix.

We calculated the number of mutated DCEX residues (direct contact residues and residues within 8Å of direct contact residues likely to influence binding) and Sum Grantham score (chemical shift).

Table S4. Mutated DC and DCEX residues and the sum Grantham Grantham score of invertebrate metazoan ACEs

Organism	# mutated DC residues	# mutated DCEX residues	Sum Grantham Score
African social velvet spider	15	35	2797
Honeybee	15	39	3083
California two-spotted octopus	9	30	2102
,Filarial nematode worm	16	45	2966
Trichoplax reptans	10	33	2464
Body louse	14	33	2561
Water flea	15	34	2895
Fruit fly	15	35	2580
African malaria mosquito	14	36	3080
Californian leech	15	36	2693
Giant owl limpet	12	28	2034

Original Wuhan-Hu-1 strain

We then modelled Wuhan-Hu-1 S-protein: invertebrate ACE using our modelling platform. We used 6m0j as the template. All the models gave acceptable nDOPE scores suggesting the models were of good quality and could be used for calculating changes in the stability of the complex (Table S5).

Table S5. Normalised DOPE scores of SARS-CoV-2 Wuhan-Hu-1 S-protein: invertebrate metazoan ACE complexes

Sequence	nDOPE score
African social velvet spider	-1.24
Honeybee	-0.89
California two-spotted octopus	-0.86
Filarial nematode worm	-0.85
Trichoplax reptans	-1.27
Body louse	-1.03
Water flea	-1.11
Fruit fly	-1.10
African malaria mosquito	-0.96
Californian leech	-1.36
Giant owl limpet	-1.39

For each complex, ΔΔG were predicted by mutating the invertebrate ACE interface residue to the appropriate residue in the human ACE2. In our previous analysis, any animals with predicted ΔΔG <= 3.72 are considered to be at risk.

Arthropod

Species	Predicted ΔΔG (DCEX)
Body louse	-1.165
Water flea	-0.530
African social velvet spider	1.152
Honeybee	1.679
Fruit fly	4.164
Giant owl limpet	5.353
African malaria mosquito	8.293

Non-Arthropod

Species	Predicted ΔΔG (DCEX)
Californian leech	-1.409
California two-spotted octopus	-0.783
Trichoplax reptans	-0.44
Filarial nematode worm	3.157

Susceptible to infection

Not susceptible to infection

Figure S6. Predicted ΔΔG values of SARS-CoV-2 Wuhan-Hu-1 S-protein: invertebrate metazoan ACE complexes for direct contact plus extended (DCEX) residues.

N501Y mutant

We then modelled N501Y S-protein: invertebrate ACE using our modelling platform. We used 6m0j as the template.

Table S6. Normalised DOPE scores of SARS-CoV-2 N501Y S-protein: invertebrate metazoan ACE complexes

Sequence	nDOPE score
African social velvet spider	-1.34
Honeybee	-0.89
California two-spotted octopus	-0.92
Filarial nematode worm	-0.86
Trichoplax reptans	-1.29
Body louse	-0.93
Water flea	-1.18
Fruit fly	-1.09
African malaria mosquito	-0.93
Californian leech	-1.26
Giant owl limpet	-1.43

For each complex, ΔΔG were predicted by mutating the invertebrate ACE interface residue to the appropriate residue in the human ACE2. In our previous analysis, any animals with predicted ΔΔG <= 3.72 are considered to be at risk.

Arthropod

Species	Predicted ΔΔG (DCEX)
Body louse	-1.921
Water flea	-1.805
African social velvet spider	0.589
Honeybee	1.839
Fruit fly	5.995
Giant owl limpet	5.43
African malaria mosquito	7.02

Non-Arthropod

Species	Predicted ΔΔG (DCEX)
Californian leech	-1.954
California two-spotted octopus	-1.784
Trichoplax reptans	-1.036
Filarial nematode worm	5.938

Susceptible to infection

Not susceptible to infection

Figure S7. Predicted ΔΔG values of SARS-CoV-2 N501Y S-protein: invertebrate metazoan ACE complexes for direct contact plus extended (DCEX) residues.

It can be seen from Figure S7 that other invertebrates also have low ΔΔG values, including several species (octopus, African spider, honeybee) that are not ectoparasites, suggesting that the stability of the SARS-Cov-2 Spike:ACE complex is not a result of selection pressure on the virus to evolve binding affinity to both animal hosts and their symbiotic insect hosts.

Supplementary Methods 1: Residue Conservation scores

To detect highly conserved residues likely to be involved in ACE2 binding to SARS-CoV-2, we scanned the receptor binding domain of implicated in the domain function against CATH domain functional families (FunFams)[1,2]. CATH FunFams group together homologous domain sequences likely to have highly similar structures and functions. They have been used in previous analyses to analyse the likely infection of animals by SARS-CoV-2 [3].

ACE2 sequences were scanned a hidden Markov model (HMM) library for CATH-Gene3D version 4.3 FunFams [1] and the best matches resolved by cath-resolve-hits [4] using a bit score cut-off of 25 and a minimum query coverage of 80%. Each hit was subsequently re-aligned to the matching FunFam using Clustal[5] in Jalview v1.8.3 [6].

We used the ScoreCons program[7] to measure conservation scores for residues in the ACE2 domain. We checked that the multiple alignment of the FunFam had sufficiently high information content to allow accurate identification of conserved residues. Information content is calculated as the diversity of positions(DOPs) score [7–9]. Highly conserved residues are identified for DOPs ≥ 70 and ScoreCons ≥ 0.7.

Supplementary Methods 2: Structure analysis

We used information from a previous study of CoV2 S:ACE2 complex to identify residues involved in the S-protein:ACE2 interface. This information had been compiled from several sources including PDBe [10], PDBsum [11]. We also examined structural evidence (e.g. from crystallography, cryo-EM and homology modelling) in the literature [12–17]]. Manual inspection of key regions of the human complex (PDB ID 6M0J), reported by earlier studies [13,18,19], was also performed using UCSF Chimera v1.15 [20].

We predicted H-bonds, salt bridges and rendered the structural images using Chimera. Side chain rotamers had been optimised using the slow refinement option in MODELLER 9.24 [21] to build the models. However, for key hot spot regions we predicted H-bonds by relaxing the allowable H-bond angle constraint. Other studies had also highlighted important sites by alanine scanning mutagenesis, deep mutagenesis experiments [13,15,17,22,23], and sites under positive selection [24,25]. These were also examined.

As with our other previous studies of binding of SARS-CoV-2 to animal ACE2 proteins [3], we also considered allosteric residues within the ACE2 DCEX set, detected using established methods: AlloSitePro [26], ENM [27] and PARS [28]. The performance of AllositePro has been endorsed by recent studies [26]. DC and DCEX sites under positive selection were detected using codon-based methods, including mixed effect model of evolution [29], available at the Datamonkey Adaptive Evolution web-server [30]. These

methods estimate dN/dS ratio for every codon in an alignment. We analysed evidence of positive selection using a codon alignment of all ACE2 orthogue sequences. Potential recombinant sequences were identified using RDP [31] version 5 and were excluded prior to selection pressure analyses.

Reference

1. Sillitoe, I.; Bordin, N.; Dawson, N.; Waman, V.P.; Ashford, P.; Scholes, H.M.; Pang, C.S.M.; Woodridge, L.; Rauer, C.; Sen, N.; et al. CATH: Increased Structural Coverage of Functional Space. *Nucleic Acids Res.* **2021**, *49*, D266–D273, doi:10.1093/nar/gkaa1079.
2. Dawson, N.L.; Sillitoe, I.; Lees, J.G.; Lam, S.D.; Orengo, C.A. CATH-Gene3D: Generation of the Resource and Its Use in Obtaining Structural and Functional Annotations for Protein Sequences. In *Protein Bioinformatics: From Protein Modifications and Networks to Proteomics*; Wu, C.H., Arighi, C.N., Ross, K.E., Eds.; Methods in Molecular Biology; Springer: New York, NY, 2017; pp. 79–110 ISBN 978-1-4939-6783-4.
3. Lam, S.D.; Bordin, N.; Waman, V.P.; Scholes, H.M.; Ashford, P.; Sen, N.; van Dorp, L.; Rauer, C.; Dawson, N.L.; Pang, C.S.M.; et al. SARS-CoV-2 Spike Protein Predicted to Form Complexes with Host Receptor Protein Orthologues from a Broad Range of Mammals. *Sci. Rep.* **2020**, *10*, 16471, doi:10.1038/s41598-020-71936-5.
4. Lewis, T.E.; Sillitoe, I.; Lees, J.G. Cath-Resolve-Hits: A New Tool That Resolves Domain Matches Suspiciously Quickly. *Bioinformatics* **2019**, *35*, 1766–1767, doi:10.1093/bioinformatics/bty863.
5. Higgins, D.G.; Sharp, P.M. CLUSTAL: A Package for Performing Multiple Sequence Alignment on a Microcomputer. *Gene* **1988**, *73*, 237–244, doi:10.1016/0378-1119(88)90330-7.
6. Waterhouse, A.M.; Procter, J.B.; Martin, D.M.A.; Clamp, M.; Barton, G.J. Jalview Version 2--a Multiple Sequence Alignment Editor and Analysis Workbench. *Bioinforma. Oxf. Engl.* **2009**, *25*, 1189–1191, doi:10.1093/bioinformatics/btp033.
7. Valdar, W.S.J. Scoring Residue Conservation. *Proteins* **2002**, *48*, 227–241, doi:10.1002/prot.10146.
8. Dessailly, B.H.; Dawson, N.L.; Mizuguchi, K.; Orengo, C.A. Functional Site Plasticity in Domain Superfamilies. *Biochim. Biophys. Acta BBA - Proteins Proteomics* **2013**, *1834*, 874–889, doi:10.1016/j.bbapap.2013.02.042.
9. Das, S.; Lee, D.; Sillitoe, I.; Dawson, N.L.; Lees, J.G.; Orengo, C.A. Functional Classification of CATH Superfamilies: A Domain-Based Approach for Protein Function Annotation. *Bioinformatics* **2015**, *31*, 3460–3467, doi:10.1093/bioinformatics/btv398.
10. Armstrong, D.R.; Berrisford, J.M.; Conroy, M.J.; Gutmanas, A.; Anyango, S.; Choudhary, P.; Clark, A.R.; Dana, J.M.; Deshpande, M.; Dunlop, R.; et al. PDBe: Improved Findability of Macromolecular Structure Data in the PDB. *Nucleic Acids Res.* **2019**, gkz990, doi:10.1093/nar/gkz990.
11. Laskowski, R.A. PDBsum: Summaries and Analyses of PDB Structures. *Nucleic Acids Res.* **2001**, *29*, 221–222, doi:10.1093/nar/29.1.221.
12. Li, Y.; Wang, H.; Tang, X.; Ma, D.; Du, C.; Wang, Y.; Pan, H.; Zou, Q.; Zheng, J.; Xu, L.; et al. Potential Host Range of Multiple SARS-like Coronaviruses and an Improved ACE2-Fc Variant That Is Potent against Both SARS-CoV-2 and SARS-CoV-1. *bioRxiv* **2020**, 2020.04.10.032342, doi:10.1101/2020.04.10.032342.
13. Lan, J.; Ge, J.; Yu, J.; Shan, S.; Zhou, H.; Fan, S.; Zhang, Q.; Shi, X.; Wang, Q.; Zhang, L.; et al. Structure of the SARS-CoV-2 Spike Receptor-Binding Domain Bound to the ACE2 Receptor. *Nature* **2020**, 1–6, doi:10.1038/s41586-020-2180-5.
14. Hussain, M.; Jabeen, N.; Raza, F.; Shabbir, S.; Baig, A.A.; Amanullah, A.; Aziz, B. Structural Variations in Human ACE2 May Influence Its Binding with SARS-CoV-2 Spike Protein. *J. Med. Virol.* **2020**, doi:10.1002/jmv.25832.
15. Melin, A.D.; Janiak, M.C.; Marrone, F.; Arora, P.S.; Higham, J.P. *Comparative ACE2 Variation and Primate COVID-19 Risk*; Genetics, 2020;
16. Procko, E. The Sequence of Human ACE2 Is Suboptimal for Binding the S Spike Protein of SARS Coronavirus 2. *bioRxiv* **2020**, 2020.03.16.994236, doi:10.1101/2020.03.16.994236.
17. Wan, Y.; Shang, J.; Graham, R.; Baric, R.S.; Li, F. Receptor Recognition by the Novel Coronavirus from Wuhan: An Analysis Based on Decade-Long Structural Studies of SARS Coronavirus. *J. Virol.* **2020**, *94*, e00127-20, /jvi/94/7/JVI.00127-20.atom, doi:10.1128/JVI.00127-20.
18. Shang, J.; Ye, G.; Shi, K.; Wan, Y.; Luo, C.; Aihara, H.; Geng, Q.; Auerbach, A.; Li, F. Structural Basis of Receptor Recognition by SARS-CoV-2. *Nature* **2020**, *581*, 221–224, doi:10.1038/s41586-020-2179-y.
19. Brielle, E.S.; Schneidman-Duhovny, D.; Linial, M. The SARS-CoV-2 Exerts a Distinctive Strategy for Interacting

with the ACE2 Human Receptor. *Viruses* **2020**, *12*, 497, doi:10.3390/v12050497.
20. Pettersen, E.F.; Goddard, T.D.; Huang, C.C.; Couch, G.S.; Greenblatt, D.M.; Meng, E.C.; Ferrin, T.E. UCSF Chimera--a Visualization System for Exploratory Research and Analysis. *J. Comput. Chem.* **2004**, *25*, 1605–1612, doi:10.1002/jcc.20084.
21. Webb, B.; Sali, A. Comparative Protein Structure Modeling Using MODELLER. *Curr. Protoc. Bioinforma.* **2016**, *54*, 5.6.1-5.6.37, doi:10.1002/cpbi.3.
22. Luan, J.; Lu, Y.; Jin, X.; Zhang, L. Spike Protein Recognition of Mammalian ACE2 Predicts the Host Range and an Optimized ACE2 for SARS-CoV-2 Infection. *Biochem. Biophys. Res. Commun.* **2020**, *526*, 165–169, doi:10.1016/j.bbrc.2020.03.047.
23. Yan, R.; Zhang, Y.; Li, Y.; Xia, L.; Guo, Y.; Zhou, Q. Structural Basis for the Recognition of SARS-CoV-2 by Full-Length Human ACE2. *Science* **2020**, *367*, 1444–1448, doi:10.1126/science.abb2762.
24. Frank, H.K.; Enard, D.; Boyd, S.D. *Exceptional Diversity and Selection Pressure on SARS-CoV and SARS-CoV-2 Host Receptor in Bats Compared to Other Mammals*; Evolutionary Biology, 2020;
25. Damas, J.; Hughes, G.M.; Keough, K.C.; Painter, C.A.; Persky, N.S.; Corbo, M.; Hiller, M.; Koepfli, K.-P.; Pfenning, A.R.; Zhao, H.; et al. Broad Host Range of SARS-CoV-2 Predicted by Comparative and Structural Analysis of ACE2 in Vertebrates. *Proc. Natl. Acad. Sci.* **2020**, 202010146, doi:10.1073/pnas.2010146117.
26. Huang, W.; Lu, S.; Huang, Z.; Liu, X.; Mou, L.; Luo, Y.; Zhao, Y.; Liu, Y.; Chen, Z.; Hou, T.; et al. Allosite: A Method for Predicting Allosteric Sites. *Bioinformatics* **2013**, *29*, 2357–2359, doi:10.1093/bioinformatics/btt399.
27. Li, H.; Chang, Y.-Y.; Lee, J.Y.; Bahar, I.; Yang, L.-W. DynOmics: Dynamics of Structural Proteome and Beyond. *Nucleic Acids Res.* **2017**, *45*, W374–W380, doi:10.1093/nar/gkx385.
28. Panjkovich, A.; Daura, X. PARS: A Web Server for the Prediction of Protein Allosteric and Regulatory Sites. *Bioinformatics* **2014**, *30*, 1314–1315, doi:10.1093/bioinformatics/btu002.
29. Murrell, B.; Wertheim, J.O.; Moola, S.; Weighill, T.; Scheffler, K.; Kosakovsky Pond, S.L. Detecting Individual Sites Subject to Episodic Diversifying Selection. *PLoS Genet.* **2012**, *8*, e1002764, doi:10.1371/journal.pgen.1002764.
30. Weaver, S.; Shank, S.D.; Spielman, S.J.; Li, M.; Muse, S.V.; Kosakovsky Pond, S.L. Datamonkey 2.0: A Modern Web Application for Characterizing Selective and Other Evolutionary Processes. *Mol. Biol. Evol.* **2018**, *35*, 773–777, doi:10.1093/molbev/msx335.
31. Martin, D.P.; Murrell, B.; Golden, M.; Khoosal, A.; Muhire, B. RDP4: Detection and Analysis of Recombination Patterns in Virus Genomes. *Virus Evol.* **2015**, *1*, doi:10.1093/ve/vev003.
